# Ketamine and transcranial magnetic stimulation treatment for bipolar II disorder: a case report

**DOI:** 10.1186/s13256-015-0520-0

**Published:** 2015-03-31

**Authors:** Steven RD Best, Brian P Griffin, Dan G Pavel

**Affiliations:** 1 The Neuroscience Center, 440 Lake Cook Road, Building 2, Deerfield, IL 60015 USA; 2 Independent Practice, 333 East Ontario Suite 1203B, Chicago, IL USA; 3 PathFinder Brain SPECT, 440 Lake Cook Road, Suite 3, Deerfield, IL 60015 USA

**Keywords:** Ketamine, Transcranial magnetic stimulation, TMS, Bipolar II

## Abstract

**Introduction:**

To the best of our knowledge, this is the first case report of successful treatment for bipolar II disorder using a combined ketamine and transcranial magnetic stimulation treatment.

**Case presentation:**

A 43-year-old Caucasian unemployed man presented to us with treatment-resistant bipolar II disorder, currently in a mixed state. A psychometric assessment and brain single-photon emission computer tomography scan were conducted at baseline. His psychometric assessment revealed severe depressive and manic symptoms that were consistent with bipolar II disorder. Findings from a brain single-photon emission computer tomography scan converged with those from his psychometric assessment. The combined ketamine and transcranial magnetic stimulation treatment was administered a total of 24 times over five months, with his ketamine dosage increased from 50mg at the first treatment to 600mg by the last. Starting after the second treatment, he reported substantial improvements in his symptoms. A follow-up psychometric assessment and brain single-photon emission computer tomography scan five months later revealed substantial blood flow increases in the previously deficient areas.

**Conclusions:**

We provide preliminary evidence for a treatment method that magnifies the therapeutic benefits of infused ketamine along with transcranial magnetic stimulation. We postulate that this may be based on an interaction at the level of the relevant cortico-thalamo-cortical circuit(s).

## Introduction

Evidence indicates that intravenous ketamine, an N-methyl-D-aspartate antagonist, is effective in reducing depressive symptoms, including those associated with bipolar depression [1-3]. By offering rapid relief that takes effect within two hours, ketamine provides an important benefit over typical antidepressant medications [4]. In a separate body of work, there is evidence that transcranial magnetic stimulation (TMS), a noninvasive technique for stimulation of the brain, can induce antidepressant and anti-manic effects. A small study suggested that the administration of ketamine prior to TMS might interfere with treatment efficacy [5]. However, another study found that abnormal function in a frontal cortico-thalamo-cortical circuit was a decisive factor in treatment resistance [6]. It was hypothesized that a way to improve the likelihood of response to ketamine infusion would be to modulate the relevant circuit during the infusion, through TMS of the medial prefrontal area that overlays the anterior cingulate cortex. It was postulated that an improvement in symptoms would occur when the combined effect entrained the electrophysiologic abnormality (abnormally slow and relatively non-responsive brain rhythms) and restored this network to a more responsive state [7,8].

## Case presentation

A 43-year-old Caucasian unemployed man presented with lifelong symptoms of depression, anxiety, and impulsive behavior. He reported struggling with intense depressed mood, substantial life stress including a divorce in progress, and the inability to hold a job due to the impairment and distress associated with his symptoms. He had received psychopharmacological and psychotherapeutic treatment for the previous six years, but reported that he had not experienced any clinically meaningful gains as a result of those treatments. His medication history included fluoxetine, sertraline, paroxetine, escitalopram, desvenlafaxine, duloxetine, levetiracetam, valproic acid, oxcarbazepine, ezogabine, aripiprazole, quetiapine fumarate, chlorpromazine, mesoridazine, and lithium. At his initial assessment, his medications included sertraline, valproic acid, and lisdexamfetamine dimesylate (this medication was not approved by our team, but rather was prescribed by his previous physician who believed his diagnosis to be attention deficit hyperactivity disorder). On the Thase and Rush Staging Model, he was at Stage III of treatment resistance, indicating failure of more than two adequate trials of distinct classes of antidepressants, as well as failure of an adequate trial with a tricyclic antidepressant.

He was given a primary diagnosis of bipolar II disorder, currently in a mixed state, based on a psychometric assessment conducted by an independent licensed clinical psychologist prior to the beginning of treatment. His assessment included the administration of a valid Personality Assessment Inventory (PAI) and the Beck Depression Inventory (BDI)-II. These measures showed severe depressive (PAI Depression (DEP) T score=79, BDI-II=36) and manic (PAI Mania (MAN) T score=74, PAI Aggression (AGG) T score=72) symptoms. Elevations in his life stress were also apparent (PAI Stress (STR) T score=86).

He was also assessed at baseline using brain single-photon emission computer tomography (SPECT) with 99mTechnetium-hexamethylpropyleneamineoxime (HMPAO). SPECT is a neuroimaging technique that shows the functional status of gray mater areas via the measurement of relative perfusion [9-12]. Results from this assessment revealed significant relative underperfusion bilaterally in multiple hemispheric areas, more accentuated in his frontal lobes, anterior cingulate and extensive underperfusion in his orbitofrontal and apico-mesial temporal areas. Marked hyperperfusion was seen in his right caudate head, cerebellar vermis, and retro-splenial posterior cingulate. Moderate hyperperfusion was apparent in his left caudate head, dorsal aspect of his posterior cingulate, and in an asymmetric thalamus. This combination of underperfused areas is compatible with multiple dysfunctions related to attention, memory, executive function, impulse control, and social interaction, while the hyperperfused areas are often found in anxiety and depression [13,14].

After informing him of the possible risks and benefits of the combined ketamine and TMS treatment, the new technique was used to treat his bipolar II disorder. He was first given two days of TMS pretreatment, which consisted of four treatments of 30 minutes each day, with 45-minute resting intervals between each treatment. Based on 14 years of observational evidence from our clinic, the TMS consisted of 40 minutes of 1 hertz continuous TMS administered at 115% of the motor threshold. The combined protocol treatment added the intravenous ketamine infusion (Ketalar^®^, manufactured by Par Sterile Products LLC, Parsippany, NJ, USA), which was administered concurrent to, and bracketed within, the middle 30 minutes of TMS; there were five minutes of TMS pre- and post-infusion. The TMS head coil (manufactured by Neotonus, Inc., Marietta, GA, USA) was positioned at the midline of his scalp to maximally stimulate the medial prefrontal area that overlays the anterior cingulate region. This area was targeted based on evidence that it is implicated in depression [15].

The protocol described above was repeated a total of 24 times over five months, at approximately one-week intervals. All stimulation levels were within published safety guidelines, and a certified registered nurse anesthetist personally managed the anesthesia aspects of this treatment at all times. The dosage of infused ketamine was gradually increased from 50mg at the first treatment (approximately 0.5mg/kg) to 600mg by the last treatment. The dose increases were based on his tolerance to higher doses and to symptom reduction, as well as on the clinical evaluation that was performed a few days after each treatment. The ketamine dose adjustment was previously described [16]. Institutional review board exemption was obtained for this report from an independent accredited agency (Sterling IRB) because the ketamine dose adjustment used in our current study has been previously described and approved.

**Figure 1:**
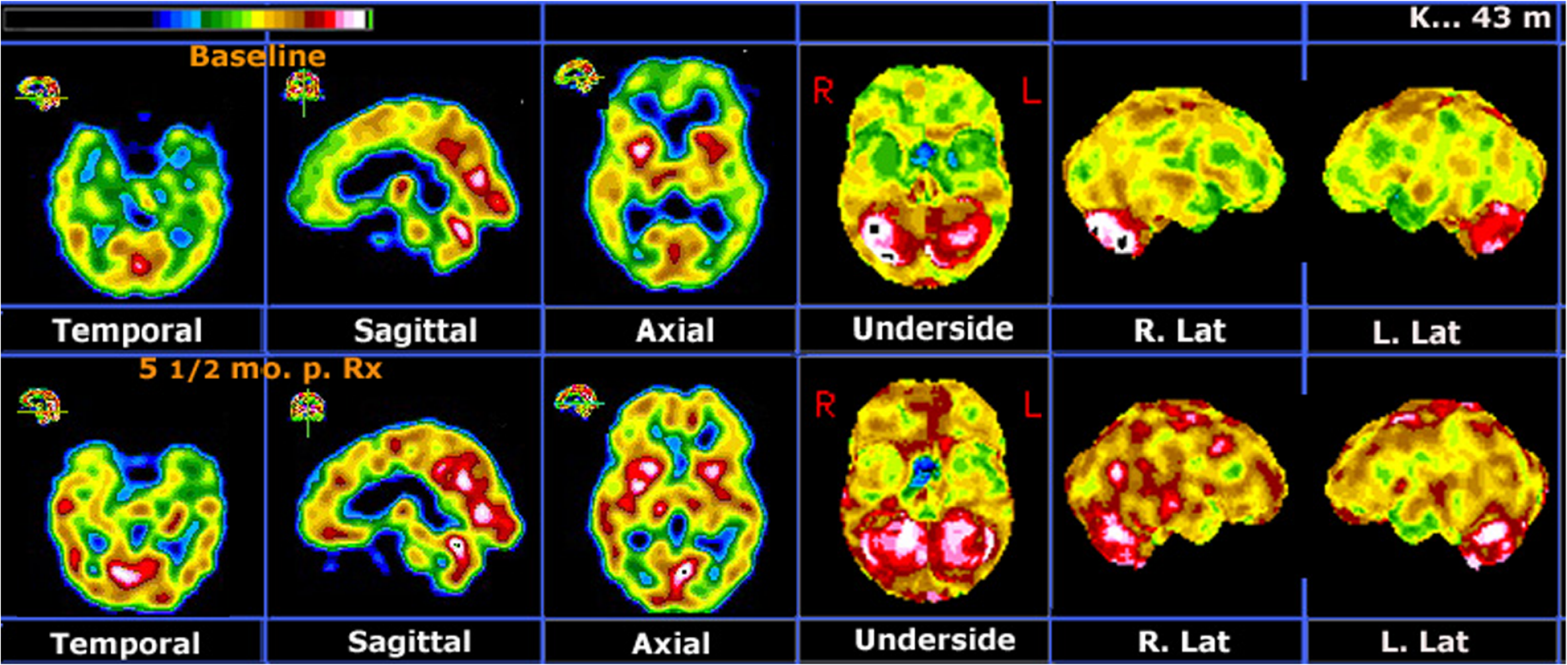
**Representative images from the various types of displays used in our protocol.** The first three images are part of the orthogonal displays and the last three are part of the stereotactic surface projections obtained with the Neurostat software (manufactured by Neurostat/3D-SSP, University of Washington, Seattle, WA, USA) [9]. Upper row: Baseline brain single-photon emission computer tomography (SPECT) shows multiple areas of hemispheric underperfusion, more accentuated in his frontal lobes, orbitofrontal areas and apico-mesial temporal areas. Marked hyperperfusion in his right putamen and in parts of his posterior cingulate and right cerebellum is also visible. Lower row: Follow-up SPECT scan at five and a half months post-treatment shows that practically all previously underperfused areas have significantly improved relative perfusion. Previously hyperperfused areas are either unchanged or increasingly hyperperfused.

Beginning after the second combined treatment, he reported substantial improvements in his symptoms and related functioning, eventually including the ability to maintain a full-time job. Five and a half months after his initial assessment, he again underwent a psychometric assessment. Results from a valid PAI and BDI-II showed substantial decreases in his symptoms related to both depression (PAI DEP T score=65, BDI-II=13) and mania (PAI MAN T score=61, PAI AGG T score=56). Remarkably, these improvements occurred even though he continued to experience elevated life stress over the duration of the treatment (PAI STR T score=82). Moreover, his follow-up brain SPECT scan showed increased relative perfusion in all previously underperfused areas (see Figure 1), thus documenting a specific functional substrate for the treatment effect.

## Conclusions

To the best of our knowledge, this is the first report of a successful treatment for bipolar II disorder using combined ketamine and TMS. Our patient reported ever-increased cognitive ability (including improved executive function) and improved affective regulation, which persisted even after the active treatment phase ended. In the future, a randomized controlled trial should be conducted to examine the efficacy of this combined treatment for the acute emotional and chronic cognitive consequences of bipolar disorders. Future work needs to further elucidate the possible mechanisms underlying combined ketamine and TMS treatment.

## Consent

Written informed consent was obtained from the patient for publication of this case report and accompanying images. A copy of the written consent is available for review by the Editor-in-Chief of this journal.

## Abbreviations

AGG: Aggression
BDI-II: Beck Depression Inventory-II
DEP: Depression
MAN: Mania
PAI: Personality Assessment Inventory
SPECT: Single-photon emission computer tomography
STR: Stress
TMS: Transcranial magnetic stimulation
